# Risk factors for vancomycin-resistant enterococcus acquisition during a large outbreak in patients aged 65 years and older

**DOI:** 10.1186/s12877-019-1398-2

**Published:** 2019-12-27

**Authors:** Benjamin Mathis, Max Haïne, Raphaële Girard, Marc Bonnefoy

**Affiliations:** 1 Departement de Geriatrie, Hopital Nord-Ouest, Trevoux, France; 2 Departement de Geriatrie, Hopital Nord-Ouest, Villefranche sur Saone, France; 3 Hospices civils de Lyon, Centre Hospitalier Universitaire Lyon Sud, Unite d’hygiene et epidemiologie, Pierre-Bénite, France; 4 Departement de Geriatrie, Hospices civils de Lyon, Centre Hospitalier Universitaire Lyon Sud, Pierre-Bénite, France

**Keywords:** Vancomycin-resistant enterococcus; older people; outbreak.

## Abstract

**Background:**

In the context of an aging population, identifying risk factors for Vancomycin-resistant enterococci (VRE), specific to older people, is important. However, if age is a known risk factor for VRE infection, a limited number of studies have focused on older patients. This study aimed to identify potential risk factors for VRE acquisition in a population aged 65 years and older, during a large VRE outbreak that occurred in a teaching hospital in Lyon, France, from December 2013 to July 2014.

**Methods:**

The present retrospective, multi-center, descriptive, and analytical study used part of a previous cohort, and included only a sub-group of patients aged 65 years and older. The analysis of the factors included in the original study was completed with factors more specific to geriatric patients. Inclusion criteria were patients aged 65 years and older, in contact with a VRE index patient. Patients were screened by rectal swabs. Univariate and multivariate logistic regression analyses were performed.

**Results:**

A total of 180 VRE contacts were included and 18 patients became carriers. Multivariate analysis showed that risk factors for VRE acquisition in older people included major contact type (RR: 5.31, 95%CI [1.33; 21.19]), number of antibiotics used (RR: 1.36, 95%CI [1.04; 1.76]), a score of McCabe = 2 (RR: 116.39, 95%CI [5.52; 2455.98]), ethylism (RR: 5.50, 95%CI [1.49; 20.25]), and dementia (RR: 7.50, 95%CI [1.89; 29.80]).

**Conclusions:**

This study was able to demonstrate risk factors for VRE acquisition in older people. These risk factors should be taken into account when in the presence of older people in a VRE infected unit.

## Background

According to a publication from the French vigilance system, the first strains of Vancomycin-resistant enterococci (VRE) were isolated in 1987–1988 in France and the United Kingdom before becoming endemic to the United States in the 1990s [1].

In France, between July 2001 and June 2015, 1140 cases of nosocomial infections involving VRE were reported [1]. VRE infectious risk is now considered a global public health issue. VRE are usually causes of morbidity, but can also be responsible for infection. In the latter case, treating the infection and limiting crossover transmission can be difficult [2]. In the context of high potential crossover transmission, each country has developed specific guidelines to control the spread of the epidemic [3, 4].

In 2013, in the USA, 66000 enterococcal infections were identified, 20,000 (30%) were VRE-related, and more than 1300 deaths were attributed to these VRE infections.

A large VRE outbreak occurred at a teaching hospital in Lyon, France, from December 2013 to July 2014. The index case was identified on December 13, 2013, detected by analysis of the liquid contained in the Kehr drain. An initial retrospective study on this outbreak was performed by Djembi et al. [5]. The study was conducted in contact patients followed during the first two months of the outbreak and identified significant factors associated with VRE acquisition. Significant risk factors were major contact, geriatric rehabilitation unit hospitalization, surgery, McCabe score equal to 2, age, hemodialysis, and central venous catheter placement.

Older people are also exposed to these bacteria and age appears as a significant risk factor in the literature. However, only a limited number of studies have focused on this population [6–8].

Based on the work provided by Djembi et al., the present study focused on identifying risk factors for VRE acquisition, during the initial period of the outbreak (December 2013 to February 2014), in a population of patients aged 65 years and older from the teaching hospitals in Lyon, France.

## Methods

### Context

A first retrospective study was published in 2017 by Djembi et al. [5] including all contact patients identified during the first two months of the outbreak. The analysis revealed factors that significantly favored the acquisition of VRE. The present study includes only patients aged 65 years or more and takes into account, in addition to the factors identified in the 2017 study, factors more specific to geriatric patients.

### Setting and population

The present study was conducted during the first two months of a *van A* VRE outbreak, which occurred in the south hospital group (SHG) of the Lyon teaching hospitals (*Hospices Civils de Lyon*, France), between December 2013 and July 2014. The study focused on the initial period of this outbreak, from December 23, 2013 to February 15, 2014.The hospitals included in the study offered about 1200 beds from Medicine, Maternity, Surgery, Emergency, Intensive Care, and Rehabilitation units. Inclusion criteria were patients aged 65 years and older, in contact with a VRE index patient, hospitalized, monitored, and detected between December 23, 2013 and February 15, 2014.

### Collection of data and definitions

Published studies on VRE have identified risk factors related to VRE acquisition. All significant factors from the literature were included, and geriatric criteria were added. Demographic variables recorded were age, sex, and unit of hospitalization. Data collected included: the number of screenings carried out until the first positive screening, which allows estimation of follow-up duration; treatments administered, such as the use of antibiotics and number of antibiotics used, Cephalosporin, Glycopeptid and Carbapenem use, Corticoid use, anti-cancer chemotherapy; associated pathologies such as diabetes with medical treatment, chronic respiratory diseases, ethylism, cognitive disorder or dementia, mental confusion, and bedsores. Data on the presence during hospital stay of digestive stomia, nasogastric tube, artificial nutrition (enteral or parenteral), urinary catheter, central venous catheter, hemodialysis, or a surgical intervention in the year before the first screening, were recorded. Information concerning the type of housing prior hospitalization (home, independent living community housing, nursing home, or long-term care), occurrence of a hospitalization stay during the previous year, and in-home health care interventions were also collected. Patient illness severity was evaluated using the McCabe score [9]. The severity index of patient condition according to disease was divided into 3 categories: 0 = non-fatal disease, 1 = fatal disease in 5 years, 2 = fatal disease in 1 year. Measures of albumin, c-reactive protein (CRP), body mass index (BMI) and a score for *activities of daily living* (ADL) [10] were recorded.

A carrier patient is defined as a patient whose test sample is positive for VRE and who may or may not be infected. An index patient is a carrier patient who is at the origin of an epidemic. A contact patient is a patient who is cared for by the same team as an index patient. Two types of contact patients are identified. Major contact concerns patients hospitalized in the unit where a carrier was present and not cared for with the appropriate complementary precautions. Minor contact relates to patients hospitalized in the unit at a time when a carrier was cared for with special precautions.

According to the national guidelines in place at the time of the epidemic, VRE carrier patients benefited from specific measures (isolation and management). Contact patients benefited from additional preventive measures in terms of contact until the end of the screening procedure. All patients were screened by a weekly rectal swab. Rectal swabs were performed by the paramedic staff of the unit and analyzed in the microbiology laboratory. The vancomycin resistant strains were tested using an E-test while polymerase chain reaction (PCR) was used to detect the *van A* gene. Results were obtained within 72 h.

### Study design

This is a retrospective, multi-center, descriptive, and analytical study using cohort data collected both retrospectively and prospectively. The analyses were carried out anonymously and the confidentiality of the data was ensured. The realization of the cohort was approved by the local ethical committee (Hospices Civils de Lyon, France) and by the National Data Protection Commission. However, according to the legislation in place at the time of the present study and because the data were collected retrospectively, this specific study did not require a novel authorization from an ethics committee or from the National Data Protection Commission.

### Statistical analysis

All statistical analyses were carried out using the IBM Statistical Package for the Social software Science (SPSS) 19.0 for Windows.

In univariate analysis, qualitative variables were compared using Chi Squared test. When available, Fisher’s exact test was preferred, otherwise Chi Squared test was used. The non-parametric Kruskal-Wallis test was used to compare continuous variables. For the multivariate analysis, a logistic regression was performed to determine risk factors for VRE acquisition. The model included factors significantly associated in univariate analysis (*p* < 0.1) and available for all patients as well as variables found to be significant in the literature. A value of *p* < 0.05 was considered statistically significant.

## Results

A total of 180 patients of mean (SD) age 79.6 (8.6) years (range: 65–103) were included in the follow-up as contacts and 18 patients became carriers. Univariate analysis revealed that significant risk factors associated with becoming a carrier patient included male sex, major contact, hospitalization in geriatric rehabilitation unit, antibiotics use, glycopeptides use, use of 3 antibiotics or more, different antibiotics number, a McCabe score equal to 2, ethylism, dementia, bedsore, albumin rate, and CRP rate (Tables 1 and 2).

**Table 1:** Characteristics of the population studied and risk factors for VRE carriage, in univariate analysis

		Total (*n* = 180)	VRE carrier patients (n = 18)		p^a^
			n	%	
Male sex		82	13	15.9	0.02
Major contact		49	9	18.4	0.03
Geriatric rehabilitation Unit hospitalization		43	11	25.6	0.02
Chemotherapy		29	1	3.4	0.17
Antibiotics		110	16	14.5	0.01
Glycopeptides		9	3	33.3	0.05
Cephalosporins		46	8	17.4	0.05
Carbapenems		9	1	11.1	0.62
Antibiotics ≥3		55	12	21.8	0.00
Corticoids		66	6	9.1	0.49
Surgery		141	14	9.9	0.58
Digestive stomia		15	2	13.3	0.46
Naso gastric tube		16	4	25	0.06
Parenteral nutrition		50	6	12	0.38
Enteral nutrition		12	2	16.2	0.34
Hemodialysis		27	4	14.8	0.27
Urinary catheter		93	12	12.9	0.14
Central venous catheter		76	10	13.2	0.17
Diabetes		61	7	11.5	0.41
Chronic respiratory pathology		26	3	11.5	0.50
McCabe = 2		3	2	66.7	0.03
In-home health care interventions		55	8	14.5	0.14
Type of housing prior hospitalization	home	168	17	10.1	0.76
	independent living community housing	3	0	0	
	long-term care	1	0	0	
	nursing home	4	0	0	
Dementia		41	9	22	0.01
Bedsore		16	5	31.3	0.01
Mental confusion		17	3	17.6	0.23
Hospitalization in the previous year		141	17	12.1	0.06
Ethylism		23	8	34.8	0.00

^a^Fisher’s exact test or Chi Squared test

**Table 2:** Continuous variables for VRE carrier and non-carrier patients, in univariate analysis

	VRE carrier patients (n = 18)	VRE non-carrier patients (*n* = 162)	p^b^
Age, years	79.39	79.67	0.62
Screening number	3.06	4.23	0.06
Number of antibiotics	3.44	1.64	0.00
Albumin rate, g.l^−1^	28.30	31.24	0.02
Body mass index, kg.m^2^	23.83	25.19	0.27
C-reactive protein rate, mg.l^−1^	35.11	20.66	0.04
Activities of daily living score	4.27	4.75	0.61

^b^Kruskal-Wallis test was used, results are given as means

In multivariate analysis, the factors significantly associated with increased risk of becoming a carrier patient were major contact as opposed to minor contact (RR: 5.31, 95%CI [1.33; 21.19]), an increase in number of antibiotics used (RR: 1.36, 95%CI [1.04; 1.76]), a McCabe score equal to 2 (RR: 116.39, 95%CI [5.52; 2455.98]), ethylism (RR: 5.50, 95%CI [1.49; 20.25]), and dementia (RR: 7.50, 95%CI [1.89; 29.80]; (Table 3).

**Table 3:** Risk factors for VRE carriage, in multivariate analysis

	Relative Risk	[95%CI]
Contact		
Minor	Reference	Reference
Major	5.31	[1.33; 21.19]
Number of antibiotics	1.36	[1.04; 1.76]
McCabe		
≤ 1	Reference	Reference
2	116.39	[5.52; 2455.98]
Ethylism		
No	Reference	Reference
Yes	5.50	[1.49; 20.25]
Dementia		
No	Reference	Reference
Yes	7.50	[1.89; 29.80]

## Discussion

The present study, performed during a large outbreak, identified for the first time risk factors for VRE carrier patients aged 65 years and older. These factors include contact type, number of antibiotics used, a McCabe score equal to 2, ethylism, and dementia.

Inpatient units and medical specialties have not been studied in detail in the context of VRE carriage. Many studies have shown a link between intensive care unit (ICU) hospitalization and VRE colonization. [11, 12]. The initial study by Djembi et al. found an association between VRE carriage and hospitalization in a geriatric rehabilitation unit. This risk factor for VRE is confirmed in the present study in univariate but not multivariate analysis. This may be explained by the choice of the study population which included only patients aged 65 years and older. Hence, geriatric unit is over-represented since all contact patients from that unit were included as they were all at least 65 years old.

Djembi et al. [5] also showed an association between major contact and VRE carriage in univariate analysis with only a trend remaining in multivariate analysis. The present study was able to confirm this association, indicating that minor contact can be considered a protective factor, and demonstrating that the recommendations and precautions put in place are of major importance to avoid VRE carriage. Patient severity is a critical criterion to be taken into account. An association between patient severity and VRE colonization has previously been reported [11, 13, 14]. However, patient severity in these studies were evaluated using either the APACHE II score (an ICU specific score) [15] or the Charlson mortality score [16]. In the current study, the McCabe score was used to allow assessment of a patient severity and not survival, contrary to the aforementioned scores [9]. Herein, a McCabe score of 2, which characterizes a fatal disease within 1 year, was found to be a risk factor for VRE carriage, thus confirming previous findings.

To our knowledge, only Djembi et al. had considered ethylism as a potential risk factor for VRE carriage. They, however, did not find a positive association between ethylism and VRE carriage in multivariate analysis, which the current study was able to show. This might be explained by the fact that regular and daily consumption of alcohol increases with age [17, 18]. It is also known that excessive consumption of alcohol can induce immunodeficiency which is also in favor of VRE acquisition.

Interestingly alcohol consumption can also impact cognitive disorders or aggravate certain neurodegenerative or vascular pathologies [19, 20], and dementia was shown herein to be the strongest risk factor for VRE acquisition. This association is shown for the first time and could also be explained by the fact that demented patients wander more often, omitting isolation procedures. In addition, antibiotics are prescribed more often in this population even without clear clinical indications [21].

Many studies have focused on the use of antibiotics and have reported a link with VRE colonization [11, 22–24]. Herein, it appeared that the risk of presenting with a positive VRE sample increased with the amount of antibiotics received. This is easily explainable by the fact that bacterial resistance is favored by the pressure of antibiotic selection thus enabling cross-transmission. Such results have previously been reported by Mc Evoy et al. [25] and Beltrami et al. [13] who also described a significant association between the number of antibiotics used and VRE colonization. Moreover, several studies in long term care residents have shown a link between VRE carriage and the use of antibiotics or previous hospitalization [7]. A large cross-sectional study focused on VRE carriage in various care settings, including acute-care hospital (ACH), intermediate-care hospital facilities (ICTF), and long-term care hospital facilities (LTCF) [8]. Prevalence was higher in ACH (14.2%) than ICTF (7.6%) or LTCF (0.8%). The common VRE acquiring risk factors between ACH and ICTF were anterior carriage, longer antibiotic duration, surgical intervention within 90 days, and the presence of skin ulcers. Independent risk factors for VRE acquisition in ACH were anterior carriage with Methicillin Resistant *Staphylococcus aureus* (MRSA), a high number of beds per room, prior use of proton pump inhibitor, and a residence time longer than 14 days. Surprisingly though, a hospital stay of more than 14 days in the ICTF was correlated with a decrease in VRE carriage.

Of important note, a study by Elizaga et al. [6] found that patients who presented with pressure sores upon admission to long term care hospitalization were at risk for VRE colonization. Although older people are at risk for pressure sores, this association was not demonstrated in the present study, likely due to the fact that skin condition was poorly described in medical records and therefore the presence of pressure sores was not systematically reported.

Regarding housing type prior to hospitalization, the literature argues that a previous stay in a nursing home is a risk factor for VRE infection [6, 26]. This, however, was not demonstrated in the present study.

A number of additional potential risk factors not reported here could have been of particular interest for the study. For instance, evaluating a patient’s direct environment could help prevent VRE colonization. Based on a literature review, Kramer et al. [27] showed that persistence of VRE could last between 5 days and 4 months on dry surfaces. Furthermore, Marci Drees et al. [28], showed that prior contamination of the hospital chamber, measured by environmental cultures and previous occupation in the previous two weeks of the chamber by patients with VRE, were factors highly predictive of VRE acquisition. At the beginning of the outbreak, samples were taken from the rooms vacated by VRE carrier patients after a hospital-grade cleaning followed by disinfection by air. However, the sampling being cumbersome and poorly tolerated by staff and patients from neighboring rooms, results could not be obtained.

Certain limits inherent to the study design are to be taken into account. The time restriction for inclusion of the initial cohort and the sub-group analysis of older people made herein restricted the number of cases available. Moreover, the retrospective nature of the study led to missing data concerning certain criteria of interest and to the inability to investigate other potential risk factors, such as environmental sampling. However, the present criteria identified during an outbreak as risk factors for older people should be taken into account when facing VRE carriage in any medical unit.

## Conclusions

The present study indicates that risk factors for VRE acquisition among people aged 65 years and older are contact type, number of antibiotics used, a McCabe score of 2, ethylism, and dementia. These risk factors should be taken into account when in the presence of older people in a VRE infected unit.

## Data Availability

The datasets generated or analyzed during the current study are not publicly available due the data copyright protection of the author’s institute, but are available from the corresponding author on reasonable request. We had a permission to collect the data from the south hospital group (SHG) of the Lyon teaching hospitals (*Hospices Civils de Lyon*, France).

## Abbreviations

VRE: Vancomycin-resistant enterococci
SHG: south hospital group
CRP: C-reactive protein
BMI: Body mass index and
ADL: Activities of daily living
PCR: Polymerase chain reaction
CNIL: Commission Nationale de l’Informatique et des Libertés
SPSS: Statistical Package for the Social software Science
ICU: Intensive care unit
ACH: Acute-care hospital
ICTF: Intermediate-care hospital facilities
LTCF: Long-term care hospital facilities
MRSA: Methicillin resistant *Staphylococcus aureus*
